# Cell Surface Polysaccharides Represent a Common Strategy for Adsorption among Phages Infecting Lactic Acid Bacteria: Lessons from Dairy Lactococci and Streptococci

**DOI:** 10.1128/mSystems.00641-21

**Published:** 2021-08-17

**Authors:** Jennifer Mahony

**Affiliations:** a School of Microbiology & APC Microbiome Ireland, University College Cork, Cork, Ireland

**Keywords:** bacteriophage, cell wall polysaccharide, receptor, virome, food fermentation, sustainable food systems, bacteriophages

## Abstract

Food fermentations rely on the application of robust bacterial starter cultures, the majority of which are represented by members of the lactic acid bacteria including Lactococcus lactis and Streptococcus thermophilus. Bacteriophage (or phage) proliferation remains one of the most significant threats to the fermentation industry. Therefore, it is imperative to define the phage ecology of fermented foods and to elucidate the mechanisms by which they recognize and bind to their bacterial hosts. Through a combination of functional and comparative genomics and structural analysis of the phage-host interactome, it is now possible to link the genotypes of strains of certain bacterial species to the chemical composition/structure of the associated cell wall polysaccharides (CWPS). In this paper, I discuss how the identification of common host recognition and binding strategies facilitates the development of rational starter culture systems and the implications of these findings in the context of sustainable food production systems.

## COMMENTARY

Fermented foods have been produced for millennia to extend the shelf life of raw foods through the competitive exclusion of many spoilage and/or pathogenic organisms while simultaneously enhancing the organoleptic attributes of the food. During food fermentations, bacterial starter cultures grow to high cell densities on dairy, plant, or meat substrates. Many of these bacterial starter cultures are members of the lactic acid bacteria (LAB), a group of Gram-positive bacteria that produce lactic acid as the primary metabolite of hexose fermentation. Decades of research pertaining to the antimicrobial activity of LAB as well as the purported “probiotic” potential of some strains have increased awareness of the potential benefits of so-called “functional foods.” Consequently, the analysis of these foods has continued to attract the attention of researchers, and their analysis has evolved from culture-based strategies to dynamic modeling using microbiological and molecular biology approaches and, more recently, through (meta)genome-based approaches. Furthermore, certain LAB and their phages have become a paradigm to understand the dynamic interplay between phages and their hosts and the role of phages in shaping the microbial landscape of fermented foods. As the food industry moves to replace many dairy- and meat-based foods with plant-based products, there is a pressing need to define the functionality of the LAB on plant-based substrates and to understand the impact of phages in such food systems. This will require focused research attention in key areas applying the lessons learned from dairy phage-host interaction research over the past decades including (i) large-scale studies of the biodiversity of LAB and phages associated with (novel) plant-based food products through metagenome/virome analysis partnered with classical culture-based approaches and (ii) elucidation of the phage-host interactome of a broad range of LAB including the lactobacilli to advance current knowledge of the range of interaction types among LAB and their phages or the conservation of infection mechanisms.

## THE “PHAGE PROBLEM”

Fermentation conditions are optimized to enhance the growth of the starter cultures; however, while the conditions are optimal for bacterial growth, the presence of high numbers of actively growing bacterial strains creates the perfect breeding ground for phage proliferation. Depending on the regularity with which specific cultures are applied and the intensity of the production regimes, phages may cause fermentation inconsistency/failure and reside in a given factory for several years (1, 2). Owing to the economic significance of the food industry and the pervasiveness of phages in fermentation facilities, the biodiversity, evolution, and interactions of lactic acid bacteria and their phages have been the subject of intense research over the past decades. Over the past decade in particular, there has been a dramatic increase in the number of available genome sequences of members of the lactic acid bacteria including Lactococcus lactis (3), Streptococcus thermophilus (4), and (formerly) *Lactobacillus* spp. (5). Similarly, the number of available phage genome sequences has risen exponentially and continues to rise. The increased availability of genome sequences has permitted an improved understanding of the genetic diversity, evolution, and functional properties of these opposing biological entities. Global phage biodiversity studies pertaining to L. lactis and S. thermophilus have highlighted the presence of dominant groups of phages for both species (6–8) while studies in the past decade have highlighted the emergence of new phage groups that have evolved through recombination with phages of the same genus/species or distinct genera (9–11). Indeed, we have established that artisanal production methods are linked to greater diversity and a higher prevalence of the so-called “rare” phage groups (12, 13). It is abundantly clear that phages continue to adapt and evolve and that the biodiversity landscape will also likely expand; therefore, consistent phage monitoring is essential.

**FIG 1:**
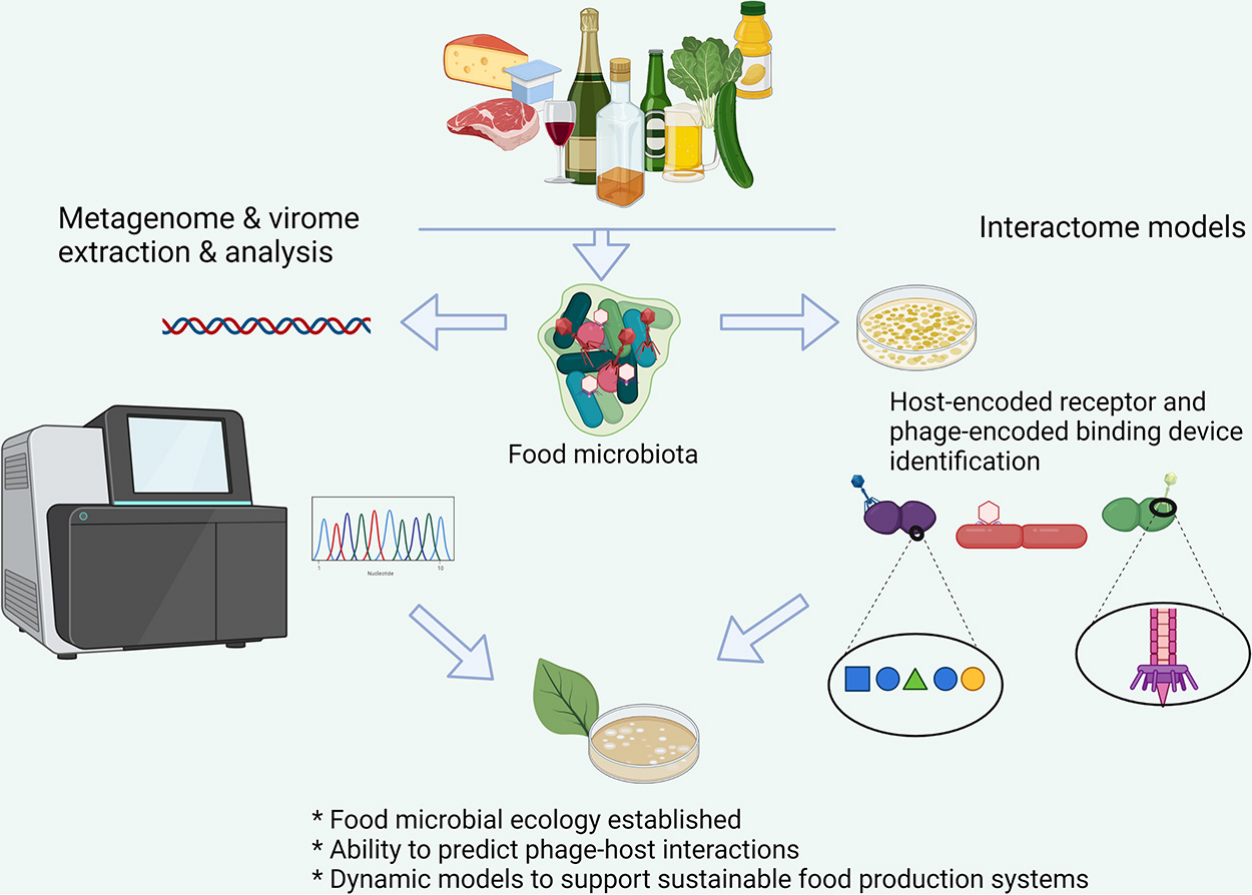
Schematic of the proposed partnership of (i) metagenome/virome analysis to establish the microbial ecology of fermented foods (left side) and (ii) culture-based and molecular biology approaches to establish interactome models to improve interaction predictions to support sustainable food production system design to reduce the risk of phage infection.

A holistic view of the biodiversity of bacterial and phage species of fermented foods is now achievable through a combination of metagenomic and virome analyses. Recently, metagenomic analysis of a range of fermented foods established that the substrate type is a major driver of the ecological diversity (14), which in turn will impact the phage population composition and dynamics. Virome studies of fermented foods are limited to date and represent a promising and informative alternative/complement to traditional phage screening studies to uncover the full extent of phage biodiversity and the emergence of novel phage species that may threaten food production systems (Fig. 1).

## UNRAVELING THE PHAGE-HOST INTERACTOME

Metagenome/virome analyses are essential to guide our understanding of the community structure of a food; however, in isolation they do not permit predictive insights into the population dynamics within the microbiota of a given food. To achieve this, mechanistic insights into the phage-host interactome are required. Using model strains and phages of both dairy lactococci and streptococci, we have identified some of the key components of the phage-host interactome in these species (15–18). These (and other) studies have identified common features and strategies employed by phages infecting these organisms including (i) the utilization of cell wall polysaccharides (CWPS) as the primary receptor and (ii) the widespread presence of readily identifiable carbohydrate-binding domains in phage-encoded structural proteins associated with distal tail structures. The locus specifying the biosynthesis of cell wall polysaccharides (CWPS) of lactococci, termed the *cwps* gene cluster, typically spans 25 to 30 kb and comprises a highly conserved 5′ end and a variable 3′ region. The 5′ conserved region specifies the biosynthetic functions for a rhamnan structure that is embedded in the peptidoglycan layer (19) while the 3′ variable region harbors genes associated with the biosynthesis of side chain glycan structures that are attached to the rhamnan component and which are exposed on the cell surface (20). The variable region of the *cwps* cluster typically harbors glycosyltransferase-encoding genes and is believed to underpin the strain-specific CWPS composition among lactococci. Comparative analysis of these clusters has permitted the identification of four genotypes (A to D) (18, 21) and eight subtypes of the C-type strains (21). Furthermore, the chemical composition and structure of representative strains have been elucidated, allowing links to be established between the gene content of these loci and the structures that they encode. For example, it is now possible to predict the (possible) number of saccharides that may contribute to the rhamnan and/or side chain polysaccharide structures. It is also possible to predict if the structures are polymerized and if component saccharides are modified, among other attributes. We have preliminary evidence that (dairy) streptococcal cell wall rhamnose-glucose (*rgp*) polysaccharide-specifying gene clusters similarly encode a two-component structure comprising a rhamnose-rich structure decorated with a saccharidic side chain; however, considerable research effort is required to decode the functions of many of the gene products of the dairy streptococcal *rgp* gene clusters. This would transform our ability to predict the types of saccharidic structures and the likelihood of interactions with specific phage groups. The model systems established for these species can now readily be adapted to a range of LAB and their phages to identify common/unique interaction and infection strategies to better understand the threat posed by phages and improve efforts to mitigate the risk of phage infection.

## HOW CAN WE PREDICT PHAGE SENSITIVITY?

The genome sequences of hundreds of lactococcal and dairy streptococcal phages are now available, providing the foundation for extensive phylogenetic analyses. Furthermore, since lactococcal and dairy streptococcal phages are well characterized, the receptor binding protein (RBP) and associated binding functions are well defined. Phylogenetic analysis of the RBPs of the dominant lactococcal 936 group phages (now termed the skunaviruses) has identified clear links to the *cwps* genotype of the partner host strain(s) (18). With the continuing increase in phage genome sequence data, it is likely that such links can also be derived for many other species of lactococcal and dairy streptococcal phages. However, before such advances can be made for other species (or genera) of LAB and their phages, it will be necessary to identify the nature of the receptor moieties and their likely diversity. A partnership of experimental and genome-based analyses is required to allow predictive models to be developed, and this represents a bottleneck in current efforts. However, the parity observed between lactococcal and streptococcal phage-host interactomes paves the way for improved functional annotations of the genomes of multiple members of the heterogenous LAB and may permit equivalent gene clusters to be identified in additional LAB species. Furthermore, with increased virome sequence data pertaining to fermented foods, it may be possible to identify and harness LAB phage genomes that are difficult to isolate and propagate and apply this reservoir of genetic information to phylogenetic analyses to establish links to host(s) receptor (genotype). The lactobacilli have recently been taxonomically reclassified, and this may improve the discernment of phage-host relationships in this complex group of LAB. This will require a concerted effort, however, since there are relatively limited phage-host studies pertaining to this group of organisms. It also represents a significant opportunity to establish the universality of phage-host interactions among LAB and their phages or, indeed, to develop additional paradigms within this biologically and economically important group of bacteria.

## CHALLENGES AND BARRIERS TO PROGRESS

While the availability of increasing numbers of genome sequences is highly beneficial to expand our understanding of the genetic complexity encompassed within a given bacterial species, it also creates challenges. As mentioned above, the former *Lactobacillus* genus has recently been reclassified and now embodies more than 20 distinct genera. Furthermore, the lactococci have also recently been reclassified such that Lactococcus cremoris has now been given species (rather than subspecies) status (22). LAB-infecting phages are typically highly specific and often infect a very limited number of strains within a given species. The taxonomic refinement of the lactococci and lactobacilli and the observed genetic diversity among species of these genera support the observed specificity of these phage-host interactions. *Lactococcus* as a genus encompasses a small number of component species, and therefore, the models that exist for saccharide-based primary phage-host interactions are likely to be representative of the majority of interactions within the genus. This is similarly likely for phage-host interactions within S. thermophilus, where a single species is represented. In contrast, the former lactobacilli represent a large number of genera, and sequence data pertaining to some genera/species within this overall cohort are limited. Furthermore, it is currently unclear if there is an equivalent to the lactococcal *cwps* and/or streptococcal *rgp* gene cluster(s) among the lactobacilli or if their interactions with phages are dependent on other saccharidic components such as teichoic acids. Similarly, among *Leuconostoc*, *Oenococcus*, and other LAB genera that are employed in food fermentations, it is unclear what types of CWPS structures they possess, and the gene clusters associated with their biosynthesis have not been described. Therefore, significant effort is required to identify these gene clusters and to analyze their architecture and composition to determine the parity of such systems, if they are present. Another bottleneck is the quality of genome annotations that are available. Many genomes deposited in public databases are not annotated to the extent that such gene clusters are readily identifiable. Furthermore, the nomenclature of genes and their predicted functions is not harmonized between strains of a species, much less between species and genera, making functional comparisons highly challenging. However, the availability of tools to improve genome annotations and protein functional predictive software will undoubtedly improve this aspect across the coming decade. Despite these challenges, there is considerable opportunity to expand knowledge of the genetic basis of phage-host interactions among LAB and to identify shared strategies as well as novel strategies and interaction types that may serve as the next generation of model systems to further improve our predictive ability.

## GOING GREEN

Dairy lactococci and streptococci have become model organisms for the study of phage-host interactions among LAB. However, while we have recently established detailed mechanistic insights into these interactions in the dairy environment, it is time to transfer that knowledge to plant-based systems. Furthermore, it will be important to define how the application of plant-based raw materials will influence the ecological landscape of the foods and the biodiversity of LAB and their phages that will thrive in this niche. To facilitate flexibility and longevity in sustainable (plant-based) food production systems, we need to learn from the lessons of the dairy environment. Phages will adapt to cultures that are available in the environment. Therefore, rational approaches to applying phage-unrelated cultures with suitable technological and organoleptic properties need to be established to allow rotations of strains and/or robust blends of strains to be applied. Using a combination of metagenome/virome sequence analysis and functional genomic/biochemical approaches, food fermentations will continue to thrive, improving the sustainability of food production.

## References

[1] Lavelle K, Murphy J, Fitzgerald B, Lugli GA, Zomer A, Neve H, Ventura M, Franz CM, Cambillau C, van Sinderen D, Mahony J . 2018. A decade of *Streptococcus thermophilus* phage evolution in an Irish dairy plant. Appl Environ Microbiol 84:e02855-17. doi:10.1128/AEM.02855-17. 29523549 PMC5930364

[2] Rousseau GM, Moineau S . 2009. Evolution of *Lactococcus lactis* phages within a cheese factory. Appl Environ Microbiol 75:5336–5344. doi:10.1128/AEM.00761-09. 19542338 PMC2725462

[3] Kelleher P, Bottacini F, Mahony J, Kilcawley KN, van Sinderen D . 2017. Comparative and functional genomics of the *Lactococcus lactis* taxon; insights into evolution and niche adaptation. BMC Genomics 18:267. doi:10.1186/s12864-017-3650-5. 28356072 PMC5372332

[4] Alexandraki V, Kazou M, Blom J, Pot B, Papadimitriou K, Tsakalidou E . 2019. Comparative genomics of *Streptococcus thermophilus* support important traits concerning the evolution, biology and technological properties of the species. Front Microbiol 10:2916. doi:10.3389/fmicb.2019.02916. 31956321 PMC6951406

[5] Zheng J, Wittouck S, Salvetti E, Franz CMAP, Harris HMB, Mattarelli P, O’Toole PW, Pot B, Vandamme P, Walter J, Watanabe K, Wuyts S, Felis GE, Gänzle MG, Lebeer S . 2020. A taxonomic note on the genus *Lactobacillus*: description of 23 novel genera, emended description of the genus *Lactobacillus* Beijerinck 1901, and union of *Lactobacillaceae* and *Leuconostocaceae*. Int J Syst Evol Microbiol 70:2782–2858. doi:10.1099/ijsem.0.004107. 32293557

[6] Lavelle K, Martinez I, Neve H, Lugli GA, Franz C, Ventura M, Bello FD, Sinderen DV, Mahony J . 2018. Biodiversity of *Streptococcus thermophilus* phages in global dairy fermentations. Viruses 10:577. doi:10.3390/v10100577. PMC6213268 30360457

[7] McDonnell B, Mahony J, Hanemaaijer L, Neve H, Noben JP, Lugli GA, Ventura M, Kouwen TR, van Sinderen D . 2017. Global survey and genome exploration of bacteriophages infecting the lactic acid bacterium *Streptococcus thermophilus*. Front Microbiol 8:1754. doi:10.3389/fmicb.2017.01754. 28955321 PMC5601072

[8] Oliveira J, Mahony J, Hanemaaijer L, Kouwen T, van Sinderen D . 2018. Biodiversity of bacteriophages infecting *Lactococcus lactis* starter cultures. J Dairy Sci 101:96–105. doi:10.3168/jds.2017-13403. 29103710

[9] McDonnell B, Mahony J, Neve H, Hanemaaijer L, Noben JP, Kouwen T, van Sinderen D . 2016. Identification and analysis of a novel group of bacteriophages infecting the lactic acid bacterium *Streptococcus thermophilus*. Appl Environ Microbiol 82:5153–5165. doi:10.1128/AEM.00835-16. 27316953 PMC4988201

[10] Mills S, Griffin C, O’Sullivan O, Coffey A, McAuliffe OE, Meijer WC, Serrano LM, Ross RP . 2011. A new phage on the ‘Mozzarella’ block: bacteriophage 5093 shares a low level of homology with other *Streptococcus thermophilus* phages. Int Dairy J 21:963–969. doi:10.1016/j.idairyj.2011.06.003.

[11] Fortier LC, Bransi A, Moineau S . 2006. Genome sequence and global gene expression of Q54, a new phage species linking the 936 and c2 phage species of *Lactococcus lactis*. J Bacteriol 188:6101–6114. doi:10.1128/JB.00581-06. 16923877 PMC1595367

[12] Mahony J, Moscarelli A, Kelleher P, Lugli GA, Ventura M, Settanni L, van Sinderen D . 2017. Phage biodiversity in artisanal cheese wheys reflects the complexity of the fermentation process. Viruses 9:45. doi:10.3390/v9030045. PMC5371800 28300778

[13] Mahony J, Randazzo W, Neve H, Settanni L, van Sinderen D . 2015. Lactococcal 949 group phages recognize a carbohydrate receptor on the host cell surface. Appl Environ Microbiol 81:3299–3305. doi:10.1128/AEM.00143-15. 25746988 PMC4407214

[14] Leech J, Cabrera-Rubio R, Walsh AM, Macori G, Walsh CJ, Barton W, Finnegan L, Crispie F, O'Sullivan O, Claesson MJ, Cotter PD . 2020. Fermented-food metagenomics reveals substrate-associated differences in taxonomy and health-associated and antibiotic resistance determinants. mSystems 5:e00522-20. doi:10.1128/mSystems.00522-20. 33172966 PMC7657593

[15] McDonnell B, Hanemaaijer L, Bottacini F, Kelleher P, Lavelle K, Sadovskaya I, Vinogradov E, Ver Loren van Themaat E, Kouwen T, Mahony J, van Sinderen D . 2020. A cell wall-associated polysaccharide is required for bacteriophage adsorption to the *Streptococcus thermophilus* cell surface. Mol Microbiol 114:31–45. doi:10.1111/mmi.14494. 32073719

[16] Legrand P, Collins B, Blangy S, Murphy J, Spinelli S, Gutierrez C, Richet N, Kellenberger C, Desmyter A, Mahony J, van Sinderen D, Cambillau C . 2016. The atomic structure of the phage Tuc2009 baseplate tripod suggests that host recognition involves two different carbohydrate binding modules. mBio 7:e01781-15. doi:10.1128/mBio.01781-15. 26814179 PMC4742702

[17] Veesler D, Spinelli S, Mahony J, Lichiere J, Blangy S, Bricogne G, Legrand P, Ortiz-Lombardia M, Campanacci V, van Sinderen D, Cambillau C . 2012. Structure of the phage TP901-1 1.8 MDa baseplate suggests an alternative host adhesion mechanism. Proc Natl Acad Sci USA 109:8954–8958. doi:10.1073/pnas.1200966109. 22611190 PMC3384155

[18] Mahony J, Kot W, Murphy J, Ainsworth S, Neve H, Hansen LH, Heller KJ, Sorensen SJ, Hammer K, Cambillau C, Vogensen FK, van Sinderen D . 2013. Investigation of the relationship between lactococcal host cell wall polysaccharide genotype and 936 phage receptor binding protein phylogeny. Appl Environ Microbiol 79:4385–4392. doi:10.1128/AEM.00653-13. 23666332 PMC3697520

[19] Sadovskaya I, Vinogradov E, Courtin P, Armalyte J, Meyrand M, Giaouris E, Palussiere S, Furlan S, Pechoux C, Ainsworth S, Mahony J, van Sinderen D, Kulakauskas S, Guerardel Y, Chapot-Chartier MP . 2017. Another brick in the wall: a rhamnan polysaccharide trapped inside peptidoglycan of *Lactococcus lactis*. mBio 8:e01303-17. doi:10.1128/mBio.01303-17. 28900021 PMC5596347

[20] Theodorou I, Courtin P, Palussiere S, Kulakauskas S, Bidnenko E, Pechoux C, Fenaille F, Penno C, Mahony J, van Sinderen D, Chapot-Chartier MP . 2019. A dual-chain assembly pathway generates the high structural diversity of cell-wall polysaccharides in *Lactococcus lactis*. J Biol Chem 294:17612–17625. doi:10.1074/jbc.RA119.009957. 31582566 PMC6873199

[21] Mahony J, Frantzen C, Vinogradov E, Sadovskaya I, Theodorou I, Kelleher P, Chapot-Chartier MP, Cambillau C, Holo H, van Sinderen D . 2020. The CWPS Rubik’s cube: linking diversity of cell wall polysaccharide structures with the encoded biosynthetic machinery of selected *Lactococcus lactis* strains. Mol Microbiol 114:582–596. doi:10.1111/mmi.14561. 32515029

[22] Li TT, Tian WL, Gu CT . 2019. Elevation of *Lactococcus lactis* subsp. *cremoris* to the species level as *Lactococcus cremoris* sp. nov. and transfer of *Lactococcus lactis* subsp. *tructae* to *Lactococcus cremoris* as *Lactococcus cremoris* subsp. *tructae* comb. nov. Int J Syst Evol Microbiol 71 (3). doi:10.1099/ijsem.0.004727. 33650946

